# Th17-to-Tfh plasticity during periodontitis limits disease pathology

**DOI:** 10.1084/jem.20232015

**Published:** 2024-05-31

**Authors:** Flora A. McClure, Kelly Wemyss, Joshua R. Cox, Hayley M. Bridgeman, Ian E. Prise, James I. King, Shafqat Jaigirdar, Annie Whelan, Gareth W. Jones, John R. Grainger, Matthew R. Hepworth, Joanne E. Konkel

**Affiliations:** 1 Lydia Becker Institute of Immunology and Inflammation, Faculty of Biology, Medicine and Health, Manchester Academic Health Science Centre, The University of Manchester, Manchester, UK; 2 Cellular and Molecular Medicine, University of Bristol, Bristol, UK

## Abstract

Th17 cell plasticity is crucial for development of autoinflammatory disease pathology. Periodontitis is a prevalent inflammatory disease where Th17 cells mediate key pathological roles, yet whether they exhibit any functional plasticity remains unexplored. We found that during periodontitis, gingival IL-17 fate-mapped T cells still predominantly produce IL-17A, with little diversification of cytokine production. However, plasticity of IL-17 fate-mapped cells did occur during periodontitis, but in the gingiva draining lymph node. Here, some Th17 cells acquired features of Tfh cells, a functional plasticity that was dependent on IL-6. Notably, Th17-to-Tfh diversification was important to limit periodontitis pathology. Preventing Th17-to-Tfh plasticity resulted in elevated periodontal bone loss that was not simply due to increased proportions of conventional Th17 cells. Instead, loss of Th17-to-Tfh cells resulted in reduced IgG levels within the oral cavity and a failure to restrict the biomass of the oral commensal community. Thus, our data identify a novel protective function for a subset of otherwise pathogenic Th17 cells during periodontitis.

## Teaser

IL-6–dependent Th17-to-Tfh plasticity during periodontitis limits disease pathology. Loss of Th17-to-Tfh cells results in altered oral antibody production and increased biomass of the oral microbial community. Our data demonstrate that Th17-to-Tfh plasticity reinforces gingival barrier integrity during inflammation.

## Graphical Abstract

**Figure:**
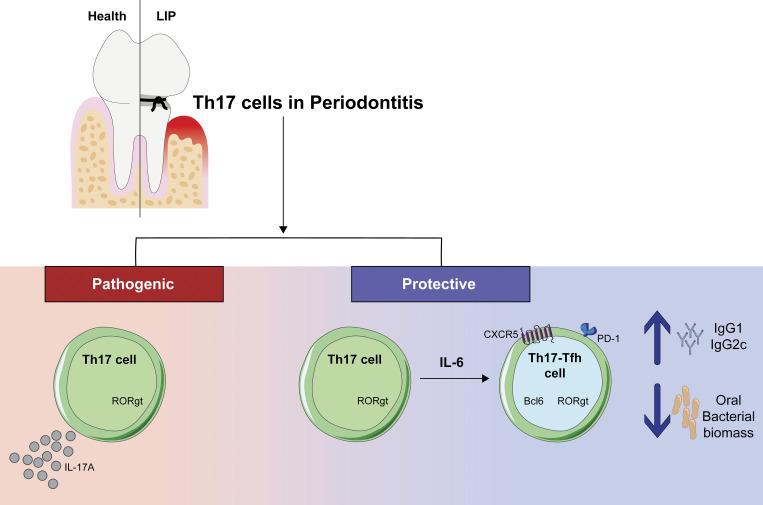

## Introduction

Compared with other mucosal barriers, immunological control at the gingiva, a key oral barrier and tooth-supporting structure, is poorly understood. This is an oversight as loss of gingival immune control results in periodontitis, the most prevalent chronic disease of humans (White et al., 2012). Periodontitis results from inappropriate dialogue between the oral microbiome and gingival immune cells, yet microbial dysbiosis alone does not precipitate periodontitis. As such detailed understanding of gingiva-specific immune networks is vital for therapeutic intervention. Additionally, periodontitis has been associated with a plethora of systemic conditions, suggested to be a potential risk factor for the development and/or exacerbation of distal diseases including rheumatoid arthritis, colitis, and Alzheimer’s disease (Hajishengallis, 2022; Konkel et al., 2019). Thus, probing the immunological drivers of periodontitis would not only promote better therapeutic options for this disease but has implications for the treatment of other conditions.

Recent work has highlighted key roles for the IL-23–IL-17 axis and T helper 17 (Th17) cells in driving periodontitis (Cardoso et al., 2009; Dutzan et al., 2016, 2018; Moutsopoulos et al., 2014; Tsukasaki et al., 2018). This has been demonstrated in preclinical models where inhibition of Th17 cell development resulted in reduced periodontitis pathology (Dutzan et al., 2018). Moreover, human cohorts with a monogenic disease that results in impaired Th17 cell development (Autosomal Dominant Hyper-IgE Syndrome; patients with loss-of-function *Stat3* mutations) exhibit not only limited gingival Th17 cells but reduced periodontal inflammation compared with age-matched healthy controls (Dutzan et al., 2018).

Although defined by their expression of the transcription factor RAR-related orphan receptor γ (RORγt) and production of IL-17A, plasticity of Th17 cells has frequently been suggested to be critical for the pathogenesis of many autoinflammatory diseases (Harbour et al., 2015; Hirota et al., 2011). Indeed, production of IFNγ by Th17 cells is reported in numerous settings, with IFNγ-expressing cells often extinguishing IL-17 production to become “ex-Th17” cells (Hirota et al., 2011; Kurschus et al., 2010; Lee et al., 2009; Muranski et al., 2011). When development of IFNγ-producing ex-Th17 cells is prevented, autoinflammatory disease pathology has been shown to be reduced (Bending et al., 2009; Lee et al., 2009; Wang et al., 2014), highlighting the importance of Th17 cell plasticity in driving aberrant inflammation. Alongside plasticity, heterogeneity within a Th17 cell population has also been outlined with pathogenic, as well as non-pathogenic, Th17 cell phenotypes described in detail (Gaublomme et al., 2015; Omenetti et al., 2019; Schnell et al., 2021). Therefore, Th17 cells are a functionally diverse population of effector T cells that acquire distinct capabilities depending on their tissue and inflammatory environment. As such, it is vital to understand the plethora of functional activities exhibited by Th17 cells if targeting these cells in autoinflammatory diseases is to be a realistic therapeutic option. Indeed, targeting IL-17 directly poses possible risks, something particularly important in the oral cavity where IL-17 controls commensal fungi and oral mucosal candidiasis development is a well-described adverse event following anti-IL-17 treatment (Davidson et al., 2021; Deodhar et al., 2019).

Here, we utilized the murine ligature-induced model of periodontitis (LIP) and IL-17A fate reporter mice (*Il17a*^*Cre*^*R26R*^*eYFP*^) to examine the functional capabilities of Th17 cells during periodontitis. Surprisingly, we found that gingival Th17 cells in LIP were largely stable with regard to their cytokine production, still dominantly making IL-17A. However, in the gingiva draining lymph node, LIP induced Th17 plasticity, driving a subset of Th17 cells to acquire T follicular helper (Tfh) features in an IL-6–dependent manner. Importantly, we outline a key role for this Th17-to-Tfh plasticity during LIP in limiting oral inflammation. When Th17 cells were prevented from acquiring a Tfh phenotype, elevated oral bacterial loads, as well as proportions of gingival neutrophils, were seen, and subsequently, periodontitis pathology was enhanced. These findings outline the complexity of Th17 functionality at the oral barrier, identifying a novel protective function for a subset of these otherwise pathogenic mediators during periodontitis.

## Results and discussion

### Minimal alteration in Th17 cell cytokine production during LIP

**Figure 1.**
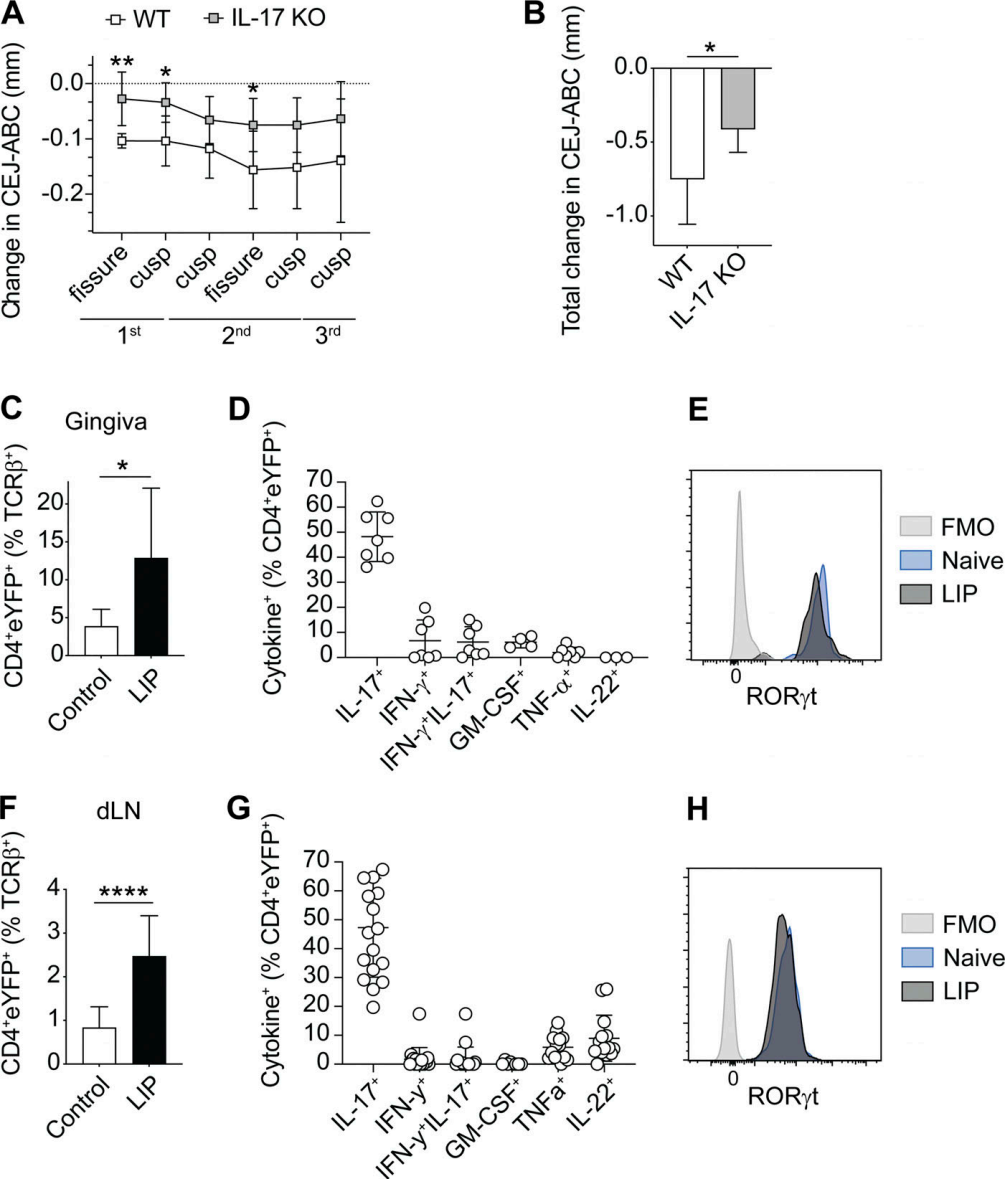
**Th17 cells continue to produce high levels of IL-17A during LIP. (A and B)** LIP was induced in IL-17^KO^ mice (Il17a^Cre-HOM^R26R^eYFP^; gray bars) or littermate controls (white bars). Bone loss was examined 10 days after ligature placement. Graphs show changes in bone heights at defined sites (A) or in total (B) determined by subtracting the CEJ-ABC distance of LIP molars from CEJ-ABC distances of un-ligated molars. Data are pooled from two separate experiments with two to four mice per group. **(C–H)** LIP was induced in *Il17a*^*Cre*^*R26R*^*eYFP*^ mice and eYFP^+^CD4^+^ T cells examined in the gingiva (C–E) and dLN (F–H) 10 days after ligature placement. **(C)** Graph shows the proportions of CD4^+^ T cells that are eYFP^+^ in the gingiva of *Il17a*^*Cre*^*R26R*^*eYFP*^ naive control (white bar) and LIP (black bar) mice. **(D)** Graph shows the proportion of cytokine^+^ eYFP^+^ CD4 T cells in the gingiva of LIP mice. **(E)** Representative histogram showing RORγt expression in CD4^+^eYFP^+^ T cells in the gingiva of naive (blue histogram) and LIP (gray histogram) mice; fluorescence minus one (FMO; light gray histogram). **(F)** Graph shows the proportion of CD4^+^ T cells that are eYFP^+^ in the dLN of *Il17a*^*Cre*^*R26R*^*eYFP*^ naive control (white bar) and LIP (black bar) mice. **(G)** Graph shows the proportion of cytokine^+^ eYFP^+^ CD4 T cells in the dLN of LIP mice. **(H)** Representative histogram showing RORγt expression in CD4^+^eYFP^+^ T cells in the dLN of naive (blue histogram) and LIP (gray histogram) mice; FMO (light gray histogram). Data are pooled from three to six experiments with two to four mice per group. *P < 0.05, **P < 0.01, ****P < 0.0001 as determined by unpaired Student’s *t* test or Mann–Whitney test (C only). Results are expressed as mean ± SD or median ± interquartile range (C only).

We first probed the requirement for IL-17A in the development of LIP. LIP severity is assessed by measuring the distance between the cemento-enamel junction (CEJ) and the alveolar bone crest (ABC) with larger distances meaning greater bone loss and pathology. In line with other publications where IL-17 signaling was limited (Dutzan et al., 2018), here, we show significantly reduced CEJ-ABC distances in IL-17A–deficient animals (Fig. 1, A and B), confirming a role for this cytokine in LIP pathology. Next, we made use of IL-17A fate-reporter (*Il17a*^*Cre*^*R26R*^*eYFP*^) mice to examine Th17 cell stability in this disease. *Il17a*^*Cre*^*R26R*^*eYFP*^ mice express Cre recombinase from the *Il17a* gene linked to enhanced yellow fluorescent protein (eYFP) expressed from the ubiquitous *Rosa26* locus; thus, any IL-17A producing cell is permanently marked as eYFP^+^ and its functions can be examined (Hirota et al., 2011). Few Th17 cells are present in healthy gingiva, yet following induction of LIP, there was a substantial expansion of IL-17A^+^CD4^+^ T cells (i.e., TCRβ^+^CD4^+^eYFP^+^ cells) in the gingiva (Fig. 1 C).

**Figure S1.**
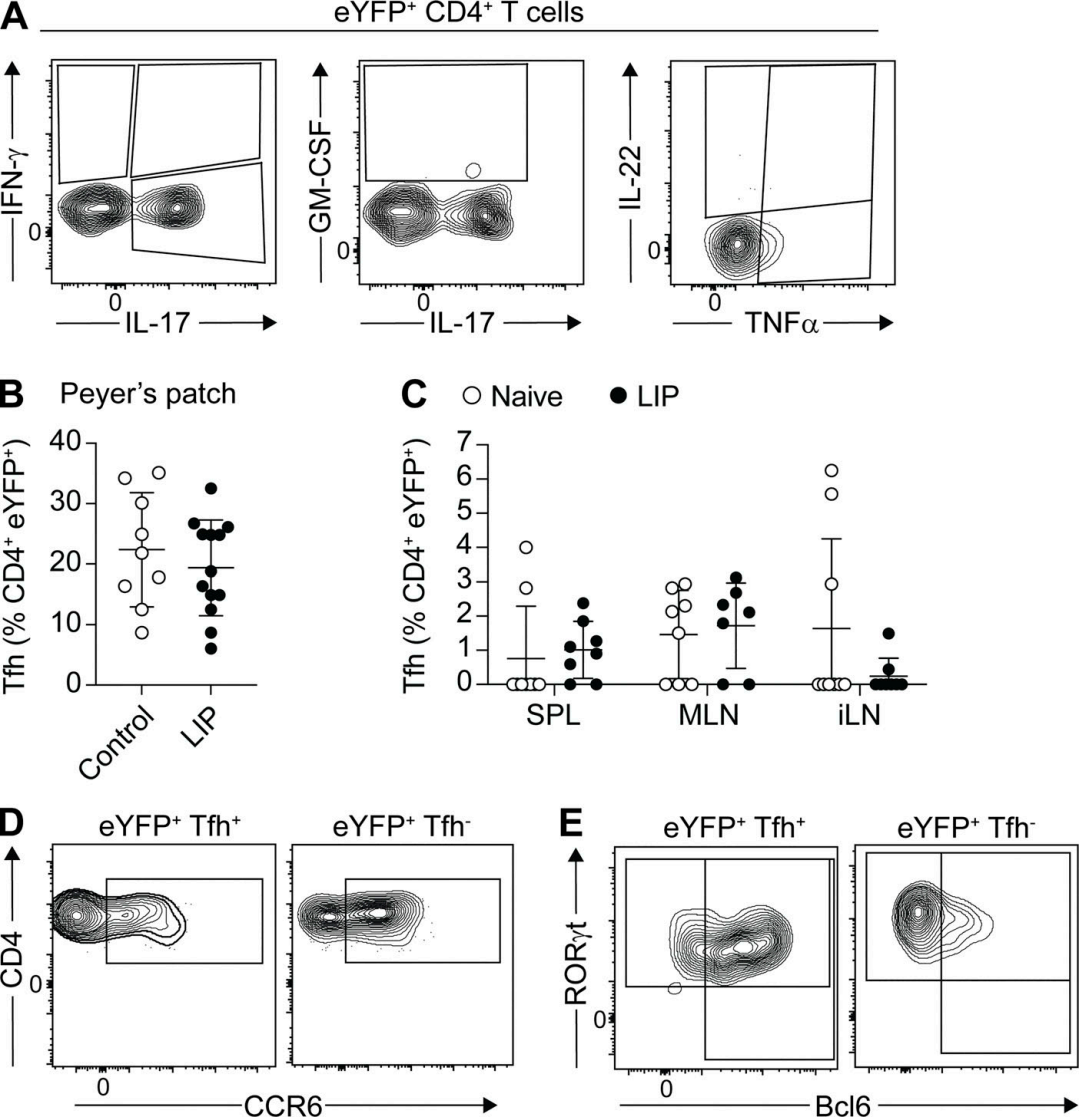
**Th17-to-Tfh plasticity occurs in the dLN during LIP. (A)** Representative FACS plots gated on eYFP^+^CD4^+^ T cells showing exemplar cytokine staining following ex vivo restimulation of cells. **(B)** Proportion of eYFP^+^ CD4^+^ T cells in the Peyer’s patches exhibiting a Tfh (CXCR5^+^PD-1^+^) phenotype in *Il17a*^*Cre*^*R26R*^*eYFP*^ mice with LIP (black circles) or naive controls (open circles). **(C)** Graph shows proportion of eYFP^+^ CD4^+^ T cells exhibiting a Tfh (CXCR5^+^PD-1^+^) phenotype in the spleen (SPL), mesenteric lymph nodes (MLN), and inguinal lymph nodes (iLN) of *Il17a*^*Cre*^*R26R*^*eYFP*^ mice with LIP or naive controls. **(D and E)** Representative FACS plots gated on eYFP^+^Tfh^+^ and eYFP^+^Tfh^−^ cells showing CCR6 (D) and RORγt and Bcl6 (E) staining. Data are pooled from three to six separate experiments with two to four mice per group. Results are expressed as mean ± SD.

Gating on total CD4^+^eYFP^+^ T cells to examine stability, ex vivo restimulation of gingiva from LIP mice demonstrated that most of these cells in the gingiva continued to singly produce IL-17A (Fig. 1 D and Fig. S1 A for example staining). Moreover, unlike in other inflammatory lesions where ex-Th17 cells making IFNγ arise (Hirota et al., 2011) or GM-CSF^+^ Th17 cells are seen (El-Behi et al., 2011), CD4^+^eYFP^+^ T cells in the gingiva did not make substantial amounts of other cytokines (Fig. 1 D). In line with this, gingiva CD4^+^eYFP^+^ T cells continued to express high levels of RORγt (Fig. 1 E). Stable production of IL-17A was also seen in the lymph nodes draining the gingiva, hereafter called dLN. Th17 cells increase in the dLN during LIP (Fig. 1 F). Upon ex vivo restimulation, total CD4^+^eYFP^+^ T cells in the dLN continued to dominantly produce IL-17A during LIP, with minimal production of other proinflammatory cytokines (Fig. 1 G) and again, these cells expressed RORγt highly (Fig. 1 H). Combined, these data indicate that during LIP, Th17 cells at the site of inflammation and in the dLN have stable production of the lineage-defining cytokine IL-17A.

### Th17-to-Tfh plasticity is seen in the gingiva draining lymph node during LIP

**Figure 2.**
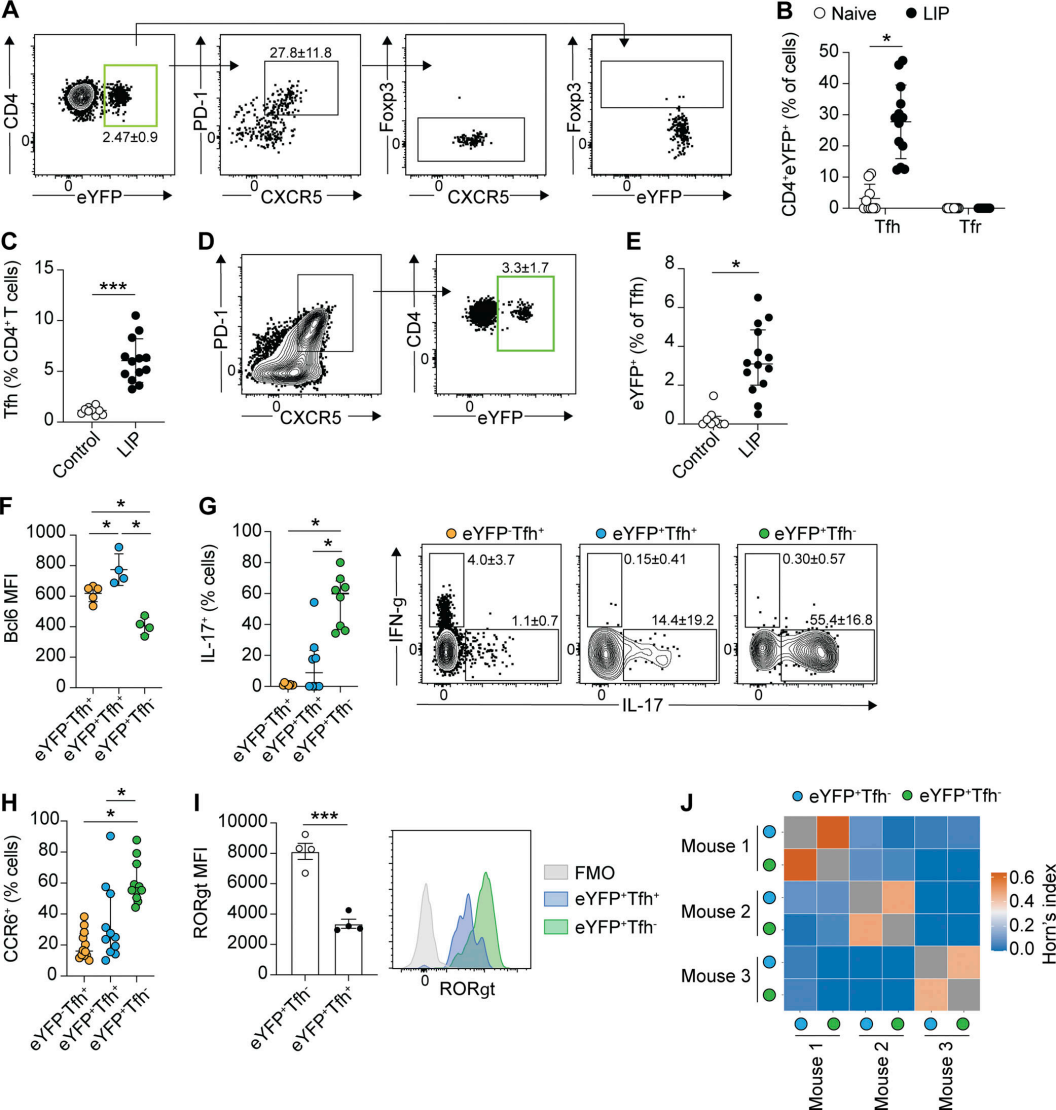
**Th17 cells exhibit a Tfh phenotype in the dLN during LIP.** LIP was induced in *Il17a*^*Cre*^*R26R*^*eYFP*^ mice and eYFP^+^CD4^+^ T cells in the dLN examined 9–10 days after ligature placement. **(A)** Representative FACS plots show the gating of eYFP^+^ CD4^+^ T cells and their Tfh (CXCR5^+^PD-1^+^) phenotype. **(B)** The proportion of eYFP^+^ CD4^+^ T cells in the dLN exhibiting a Tfh (CXCR5^+^PD-1^+^) or Tfr (CXCR5^+^PD-1^+^Foxp3^+^) phenotype in naive and LIP mice. **(C)** The proportion of total Tfh in the dLN in naive control and LIP mice. **(D and E)** Representative FACS plots and bar graph showing Tfh cells in the dLN expressing eYFP^+^ during LIP. **(F–H)** Graphs show the expression of Bcl6 (F), IL-17 (G), and CCR6 (H) in eYFP^−^Tfh^+^ (orange symbol), eYFP^+^Tfh^+^ (blue symbol), and eYFP^+^Tfh^−^ (green symbol) cells in the dLN of LIP mice. **(I)** Representative histogram of RORγt expression and bar graph showing mean fluorescence intensity (MFI) of RORγt staining in eYFP^+^Tfh^−^ (open symbol; green histogram) and eYFP^+^Tfh^+^ (black symbol; blue histogram) T cells in the dLN of LIP mice. **(J)** eYFP^+^Tfh^+^ (blue symbol) and eYFP^+^Tfh^−^ (green symbol) cells were FACS sorted from the dLN of LIP mice and TCRα chain sequencing undertaken. Repertoire analysis shows Horn’s index of similarity between eYFP^+^Tfh^+^ and eYFP^+^Tfh^−^ cells. Data are pooled from three to six experiments with two to four mice per group, apart from J where cells were sorted from three separate mice. *P < 0.05, ***P < 0.0001 as determined by unpaired Student’s *t* test (C and I), one-way ANOVA (F), Mann–Whitney (B and E), or Kruskal–Wallis (G and H) tests. Results are expressed as mean ± SD (C, F, and I) or median ± interquartile range (B, E, G, and H).

Although the total Th17 cell population in the dLN retained RORγt expression and IL-17A production, we saw that a subset of CD4^+^eYFP^+^ T cells acquired additional effector characteristics in this lymph node during LIP. Following induction of LIP, ∼30% of CD4^+^eYFP^+^ T cells in the dLN exhibited a Tfh cell–like phenotype, expressing both PD-1 and CXCR5 (Fig. 2, A and B). These CD4^+^eYFP^+^PD-1^+^CXCR5^+^ T cells did not express Foxp3 (Fig. 2, A and B) and therefore were not T follicular regulatory (Tfr) cells.

Th17-to-Tfh conversion has previously been demonstrated in Peyer’s patches under homeostatic conditions (Hirota et al., 2013), yet this was unchanged following induction of LIP (Fig. S1 B). Tfh expansion is a component of inflammatory responses, and indeed elevated proportions of total Tfh cells were seen in the dLN during LIP (Fig. 2 C). Of these expanded Tfh, only ∼3% were eYFP^+^ (Fig. 2, D and E) yet almost 30% of the CD4^+^eYFP^+^ T cells in the dLN exhibit this phenotype (Fig. 2, A and B). Although this Th17-to-Tfh cell plasticity occurred in the dLN during LIP, it was not seen in the spleen, another organ in which Th17 cells expand during LIP, or in non-draining lymph nodes (Fig. S1 C).

The population of CD4^+^eYFP^+^PD-1^+^CXCR5^+^ T cells identified in the dLN during LIP expressed the canonical Tfh transcription factor Bcl6 (Fig. 2 F). Indeed, Bcl6 was highly expressed by CD4^+^eYFP^+^PD-1^+^CXCR5^+^ (hereon termed eYFP^+^Tfh^+^) cells, with eYFP^+^Tfh^+^ exhibiting elevated Bcl6 expression compared with conventional eYFP^−^Tfh^+^ cells (Fig. 2 F). However, significantly fewer eYFP^+^Tfh^+^ cells stained positive for IL-17A upon ex vivo restimulation compared to eYFP^+^Tfh^−^ cells (eYFP^+^CD4^+^ T cells negative for CXCR5 and PD-1) (Fig. 2 G). Moreover, eYFP^+^Tfh^+^ cells had reduced expression of CCR6 (Fig. 2 H and Fig. S1 D for example staining) and RORγt (Fig. 2 I) compared with eYFP^+^Tfh^−^ cells. Thus, eYFP^+^Tfh^+^ cells expressed both Bcl6 and RORγt (Fig. S1 E), although RORγt expression was reduced compared with eYFP^+^Tfh^−^ cells. Yet, TCR sequencing revealed significant shared TCRα chain usage between eYFP^+^Tfh^+^ and eYFP^+^Tfh^−^ cells, reinforcing their relatedness (Fig. 2 J), although as expected little TCR sharing was seen between mice. Combined, these data demonstrate that LIP can drive Th17 cells in the dLN toward a Tfh cell fate, with the emerging eYFP^+^Tfh^+^ cells exhibiting key features of Tfh cells.

### Th17-to-Tfh plasticity occurs following oral inflammation and is driven by IL-6

**Figure S2.**
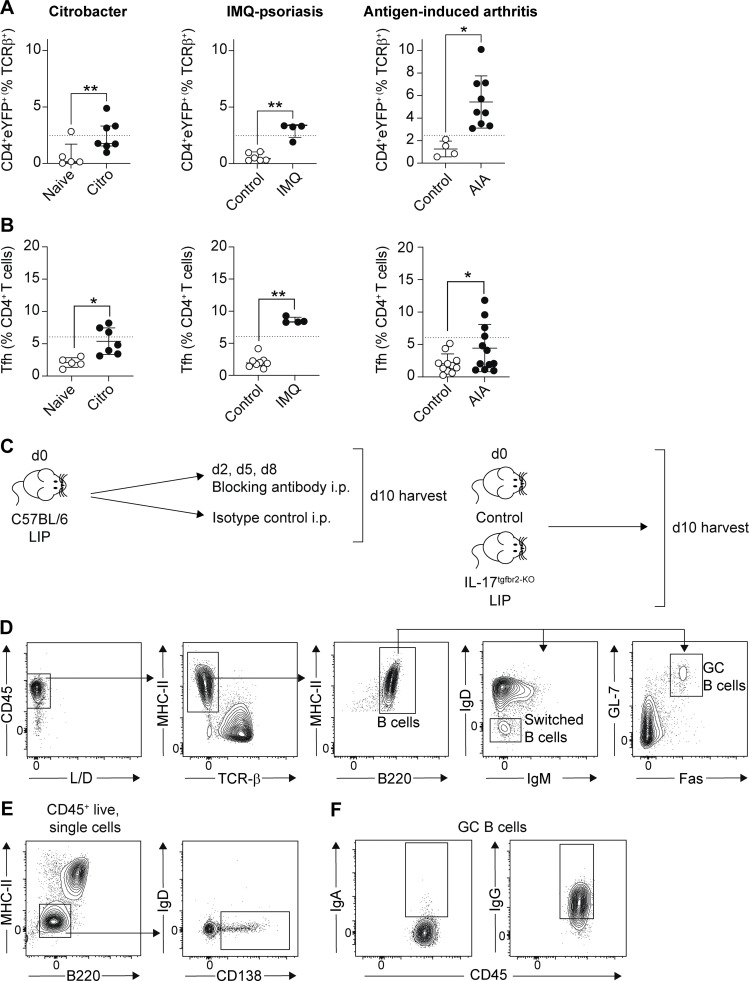
**Th17-to-Tfh plasticity is not a generic feature of all Th17-driven inflammatory responses. (A and B)** *C. rodentium* (*Citro*: left column), IMQ-induced psoriasis (IMQ: central column), and AIA (right column) were induced in *Il17a*^*Cre*^*R26R*^*eYFP*^ mice and eYFP^+^ and eYFP^−^ CD4^+^ T cells examined at the peak of disease in the appropriate draining lymph nodes. **(A)** Graphs show proportion of eYFP^+^ CD4^+^ T cells. **(B)** Graphs show proportion of eYFP^−^ Tfh T cells. Dashed lines on graphs indicate percent of cells in the dLN of LIP mice. Data are pooled from two to four experiments with two to four mice per group. **(C)** Experimental outline of LIP experiments in which TGFβ, primary IFN, or IL-6 signals were inhibited. **(D–F)** Representative FACS plots showing gating strategy and exemplar staining for B cell populations (D), PC (E), and Ig staining (F). *P < 0.05 as determined by unpaired Student’s *t* test and **P < 0.05 as determined by Mann–Whitney test. Results are expressed as mean ± SD (*, Student's *t* test) or median ± interquartile range (**, Mann–Whitney test).

**Figure 3.**
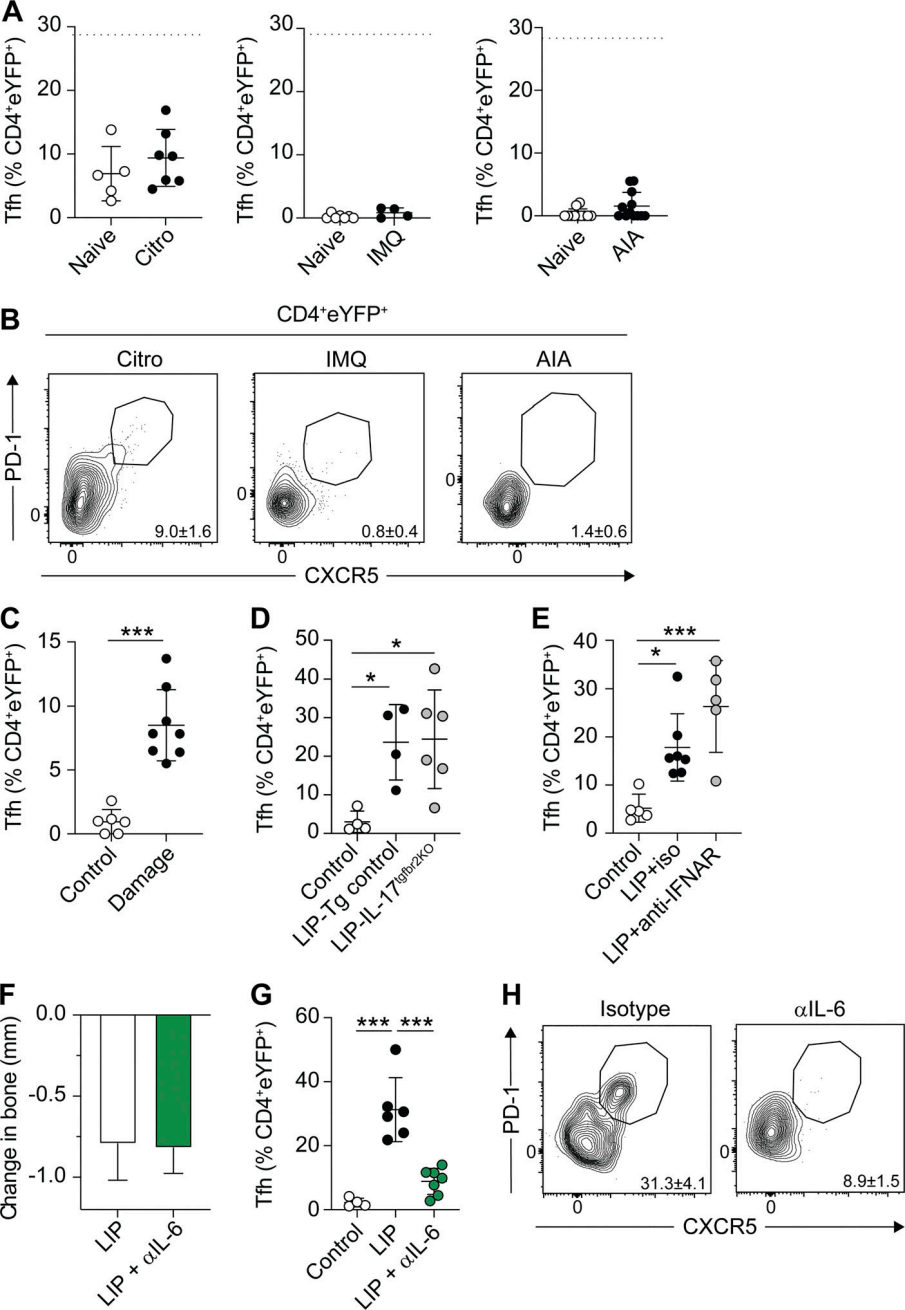
**Th17-to-Tfh plasticity is driven by IL-6. (A and B)** *C. rodentium* (*Citro*: left column), IMQ-induced psoriasis (IMQ: central column), and AIA (right column) were induced in *Il17a*^*Cre*^*R26R*^*eYFP*^ mice and eYFP^+^CD4^+^ T cells examined at the peak of disease in the appropriate draining lymph nodes. Graphs and representative FACS plots showing the proportion of eYFP^+^CD4^+^ T cells exhibiting a Tfh (CXCR5^+^PD-1^+^) phenotype. Data are pooled from two to four experiments with two to four mice per group. Dashed lines on graphs indicate the percent of cells in the dLN of LIP mice. **(C)** *Il17a*^*Cre*^*R26R*^*eYFP*^ mice experienced gingival damage every other day for 11 days, then eYFP^+^CD4^+^ T cells were examined in the dLN. Graph shows the proportion of eYFP^+^CD4^+^ T cells that have a Tfh phenotype. Data are pooled from two experiments with three to four mice per group. **(D)** LIP was induced in IL-17^TGFbRII-KO^ (Tgfbr2^fl/fl^x*Il17a*^*Cre*^*R26R*^*eYFP*^; gray symbols) and littermate control (Tgfbr2^fl/+^x*Il17a*^*Cre*^*R26R*^*eYFP*^ or Tgfbr2^+/+^x*Il17a*^*Cre*^*R26R*^*eYFP*^; black symbols) mice that were examined 9–10 days after ligature placement. Graph shows the proportion of eYFP^+^CD4^+^ T cells that have a Tfh phenotype. Data are pooled from two experiments with two to four mice per group. **(E)** LIP was induced in *Il17a*^*Cre*^*R26R*^*eYFP*^ mice, and on days 2, 5, and 8 following induction mice received either isotype control (black symbols) or anti-IFNAR (gray symbols) i.p. before being examined at day 10. Graph shows proportion of eYFP^+^ CD4^+^ T cells that have a Tfh phenotype. Data are pooled from two experiments with two to four mice per group. **(F–H)** LIP was induced in *Il17a*^*Cre*^*R26R*^*eYFP*^ mice, and on days 2, 5, and 8 following induction mice received either isotype control (black bar/symbols) or anti-IL-6 (green bar/symbols) i.p. before being examined at day 10. **(F)** Graph shows total change in bone heights in LIP mice compared with un-ligated controls. **(G and H)** Graph and representative FACS plots show proportion of eYFP^+^CD4^+^ T cells that have a Tfh phenotype. Data are pooled from two to three experiments with one to three mice per group. *P < 0.05, ***P < 0.0001 as determined by unpaired Student’s *t* test (C), one-way ANOVA (D and G), or Kruskal–Wallis (E) test. Results are expressed as mean ± SD (A, C, D, F, and G) or median ± interquartile range (E).

Next, we queried whether Th17-to-Tfh plasticity was a common feature of Th17-driven disease pathologies, as enrichment of a Tfh transcriptional signature has previously been observed in disease-driving Th17 cells in the meninges during experimental autoimmune encephalomyelitis (EAE) (Hartlehnert et al., 2021). We examined CD4^+^eYFP^+^ T cell plasticity in the imiquimod (IMQ) model of psoriasis, antigen-induced arthritis (AIA), and following *Citrobacter rodentium* infection, diseases in which increases in both eYFP^+^ and eYFP^−^Tfh^+^ cells are seen in the respective draining lymph nodes (Fig. S2, A and B). Strikingly, a population of eYFP^+^Tfh^+^ cells did not emerge in the dLN in any of these inflammatory settings (Fig. 3, A and B), indicating that acquisition of a Tfh phenotype by Th17 cells was not a default pathway occurring in all settings where Th17 cells are enriched. Thus, only specific sites and/or inflammatory contexts can drive Th17 cells to adopt a Tfh fate.

We have previously shown that chronic low-level damage to the gingiva drives expansion of Th17 cells at this site (Dutzan et al., 2017). We next examined whether the immune response induced in response to oral damage would also promote Th17-to-Tfh plasticity in the dLN. To do this, the gingiva of *Il17a*^*Cre*^*R26R*^*eYFP*^ reporter mice was damaged every other day for 11 days before dLN eYFP^+^CD4^+^ T cells were examined. Acute increases in gingival damage also led to the emergence of eYFP^+^Tfh^+^ cells in the gingiva dLN (Fig. 3 C), although to lower levels than those induced by LIP. Combined these data indicate that Th17-to-Tfh plasticity preferentially occurs within the lymph node draining the gingiva in response to oral inflammation.

We next explored the factors that support Th17 cells to acquire a Tfh phenotype in the gingiva dLN. We examined the roles of candidate factors reported to be involved in Tfh generation including TGFβ (Marshall et al., 2015; Schmitt et al., 2014), type-I IFN signaling (Cucak et al., 2009; De Giovanni et al., 2020), and IL-6 (Choi et al., 2013; Nurieva et al., 2009). This was examined utilizing antibody blockade (to inhibit IFNAR and IL-6 signals) or transgenic animals in which *tgfbr2* was specifically deleted on IL-17A–producing cells (*Tgfbr2*^fl/fl^x*Il17a*^*Cre*^*R26R*^*eYFP*^) (Fig. S2 C). Loss of TGFβ or type-I IFN signals had no effect on the development of eYFP^+^Tfh^+^ cells (Fig. 3, D and E). Administration of IL-6 blocking antibodies during the development of LIP did not impact overall periodontitis severity (Fig. 3 F). However, blockade of IL-6 signals did lead to a significant decrease in the proportion of eYFP^+^Tfh^+^ cells that emerged in the dLN during LIP (Fig. 3, G and H). Combined, these data indicate that the gingiva dLN is an environment that drives Th17-to-Tfh plasticity in an IL-6–dependent manner.

### LIP induces a B cell response skewed toward IgG

The primary function of Tfh cells is to provide crucial signals to B cells permitting their proliferation, differentiation, and affinity maturation, leading to the development of antibody-secreting B cells and plasmablasts. B cells, B cell survival factors, and antibody are all increased in the gingiva of periodontitis patients (Abe et al., 2015; Thorbert-Mros et al., 2015; Williams et al., 2021). B cells have also been shown to be drivers of pathology in preclinical periodontitis models (Abe et al., 2015; Oliver-Bell et al., 2015), yet the role of antibody and Tfh in this disease remains less well-defined. Given the unique emergence of eYFP^+^Tfh^+^ cells in the dLN during LIP, we next explored the B cell response in the dLN during periodontitis.

**Figure 4.**
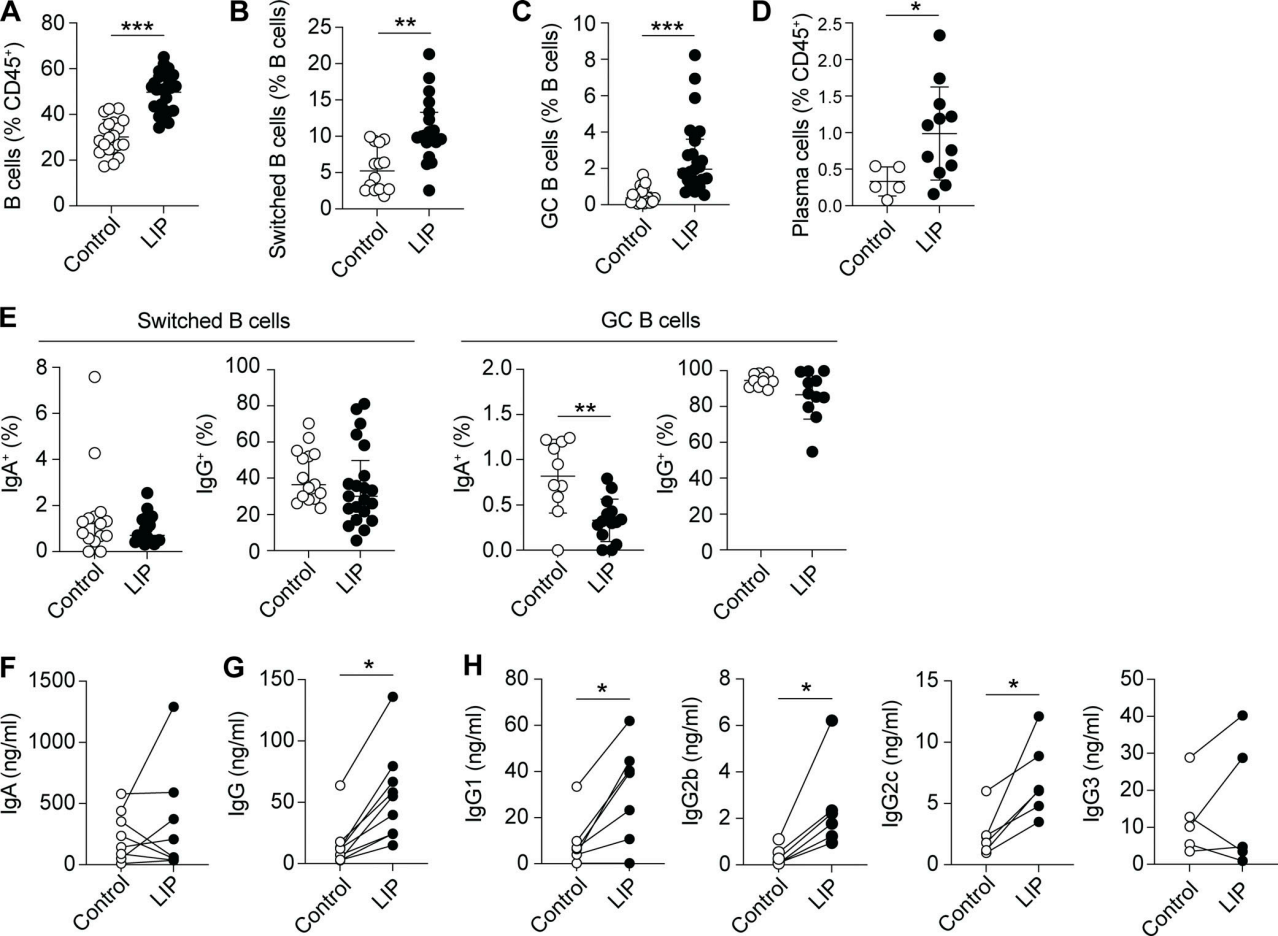
**LIP induces B cell responses and increases in oral antibody.** LIP was induced and B cell responses in the dLN and levels of antibody in oral rinses examined 9–10 days after ligature placement. **(A–C)** Bar graphs show proportions of B cells (A), switched B cells (B; IgD^−^IgM^−^), and GC B cells (C; GL7^+^Fas^+^) in the dLN of naive control (open symbols) and LIP (black symbols) mice. Data are pooled from eight experiments with two to four mice per group. **(D)** Bar graph shows proportion of PCs (CD45^+^B220^−^MHCII^−^CD138^+^) in the gingiva of naive control and LIP mice. Data are pooled from three experiments with two to four mice per group. **(E)** Graphs show expression of IgA and IgG on switched B cells (left) and GC B cells (right). Data are pooled from four to six experiments with two to four mice per group. **(F and G)** Graphs show concentration of total IgA (F) and total IgG (G) in the oral rinse of naive control or LIP mice. **(H)** Graphs show concentration of IgG1, IgG2b, IgG2c, and IgG3 in the oral rinse of naive control or LIP mice. Lines join dots from the same experiment. Data are pooled from five to eight experiments with two to four mice per group. *P < 0.05, **P < 0.005, ***P < 0.0001 as determined by unpaired Student’s *t* test (A, D, and E) or Mann–Whitney test (B, C, G, and H). Results are expressed as mean ± SD (A, D, and E) or median ± interquartile range (B, C, G, and H).

Following induction of LIP there was an increase in the frequency of B cells within the dLN and, of those, significantly more class-switched B cells and germinal center (GC) B cells (Fig. 4, A–C). Corresponding increases in plasma cells (PC) were seen in the gingiva during LIP (Fig. 4 D and Fig. S2, D–F for example staining). We examined expression of IgA and IgG on these B lineages, as IgA, followed by IgG, are the main antibody isotypes found in the murine oral cavity (Zubeidat et al., 2023). As IgA predominately originates from the salivary gland (Brandtzaeg et al., 1991; Zubeidat et al., 2023), there were low levels of IgA class-switched B cells. In contrast, we observed high levels of IgG class-switched B cells; however, the proportion of cells positive for IgG was unchanged by LIP (Fig. 4 E). These data are in line with that from periodontitis patients in which IgG deposition in the gingiva is increased during disease (Nikolopoulou-Papaconstantinou et al., 1987; Toto et al., 1978) and combined indicate that LIP induces a B cell response in the dLN dominated by IgG production.

To further explore antibody production within the oral cavity during LIP, we examined antibody levels in oral rinses by ELISA. There were no differences in IgA levels between control and LIP groups; however, IgG levels were significantly increased in the oral rinse of LIP mice (Fig. 4, F and G). Examining IgG subclasses, ELISA of oral rinses indicated that IgG3 levels were unaffected by LIP but IgG1, IgG2b, and IgG2c were all significantly increased in the oral rinse from LIP mice compared with controls (Fig. 4 H). Altogether these data reinforce a dominant role for IgG production during periodontitis and furthermore highlight a role for IgG1, IgG2b, and IgG2c in the response to oral inflammation.

### eYFP^+^Tfh^+^ cells limit LIP pathology

**Figure 5.**
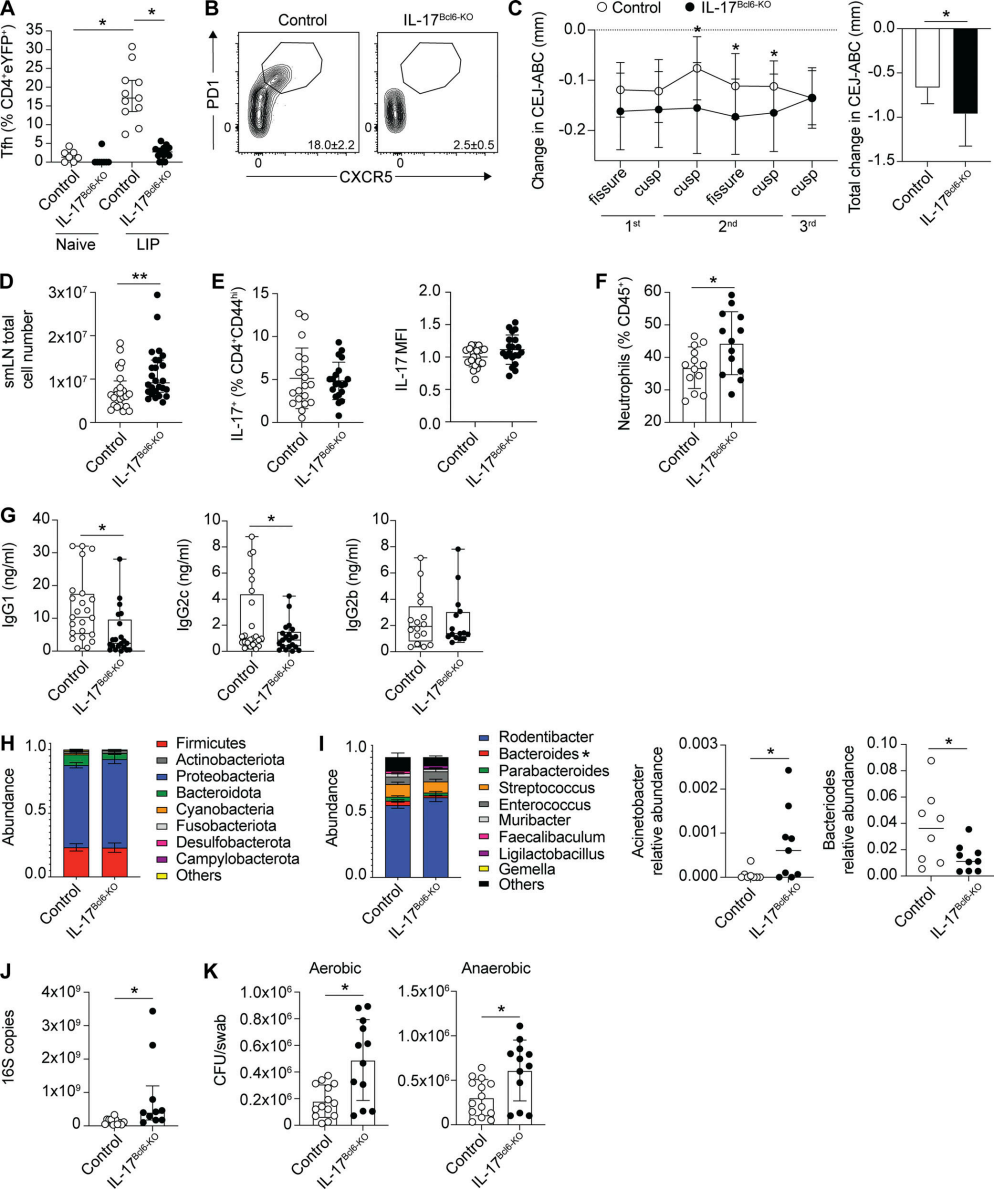
**Lack of Th17-to-Tfh plasticity during LIP enhances periodontal pathology.** LIP was induced in IL-17^Bcl6-KO^ and littermate control mice and immune parameters examined 9–10 days after ligature placement. **(A and B)** Graph (A) and representative FACS plots (B) showing the proportion of eYFP^+^CD4^+^ T cells that exhibit a Tfh (CXCR5^+^PD-1^+^) phenotype in the dLN of IL-17^Bcl6-KO^ (black symbols) and control (open symbols) mice. **(C)** CEJ-ABC distance was measured at defined points across the molars and change in bone heights determined in IL-17^Bcl6-KO^ and control mice by comparing with un-ligated molars. Bar graph shows total change in bone heights in LIP mice compared with un-ligated controls. **(D)** Graph shows total cellularity of the dLN of IL-17^Bcl6-KO^ and control mice with LIP. **(E)** Graphs show (left) the proportion of CD44^hi^CD4^+^ T cells staining positive for IL-17A and (right) MFI of that IL-17A staining normalized to MFI in control mice from the same experiment. **(F)** Graphs show the percent of neutrophils in the gingiva of IL-17^Bcl6-KO^ and control mice with LIP. **(G)** Graphs show the concentration of IgG1, IgG2c, and IgG2b in the oral rinse of IL-17^Bcl6-KO^ and control mice with LIP; box and whisker graphs show minimum to maximum values. **(H and I)** Graphs showing oral microbiome composition at the phyla (H) and genus (I) levels in control and IL-17^Bcl6-KO^ LIP animals elucidated by 16S rRNA sequencing of oral swab–derived bacteria; asterisk indicates significantly different genera. Data from *n* = 8 control samples and *n* = 9 IL-17^Bcl6-KO^ samples. **(J and K)** Graph shows total bacterial load in the oral cavity of IL-17^Bcl6-KO^ and control mice with LIP as determined by (J) 16S rRNA-based real-time PCR assay and (K) CFU enumeration from aerobic and anaerobic culture. Apart from 16S sequencing data, data are pooled from *n* = 10–26 mice per group (LIP mice) or *n* = 5–8 mice per group (naive mice) from three to seven different experiments. *P < 0.05, **P < 0.01, as determined by unpaired Student’s *t* test (C, F, and K), Mann–Whitney test (D, G, and J), or Kruskal–Wallis test (A). Results are expressed as mean ± SD (C, F, H, and I) or median ± interquartile range (A, D, and J).

**Figure S3.**
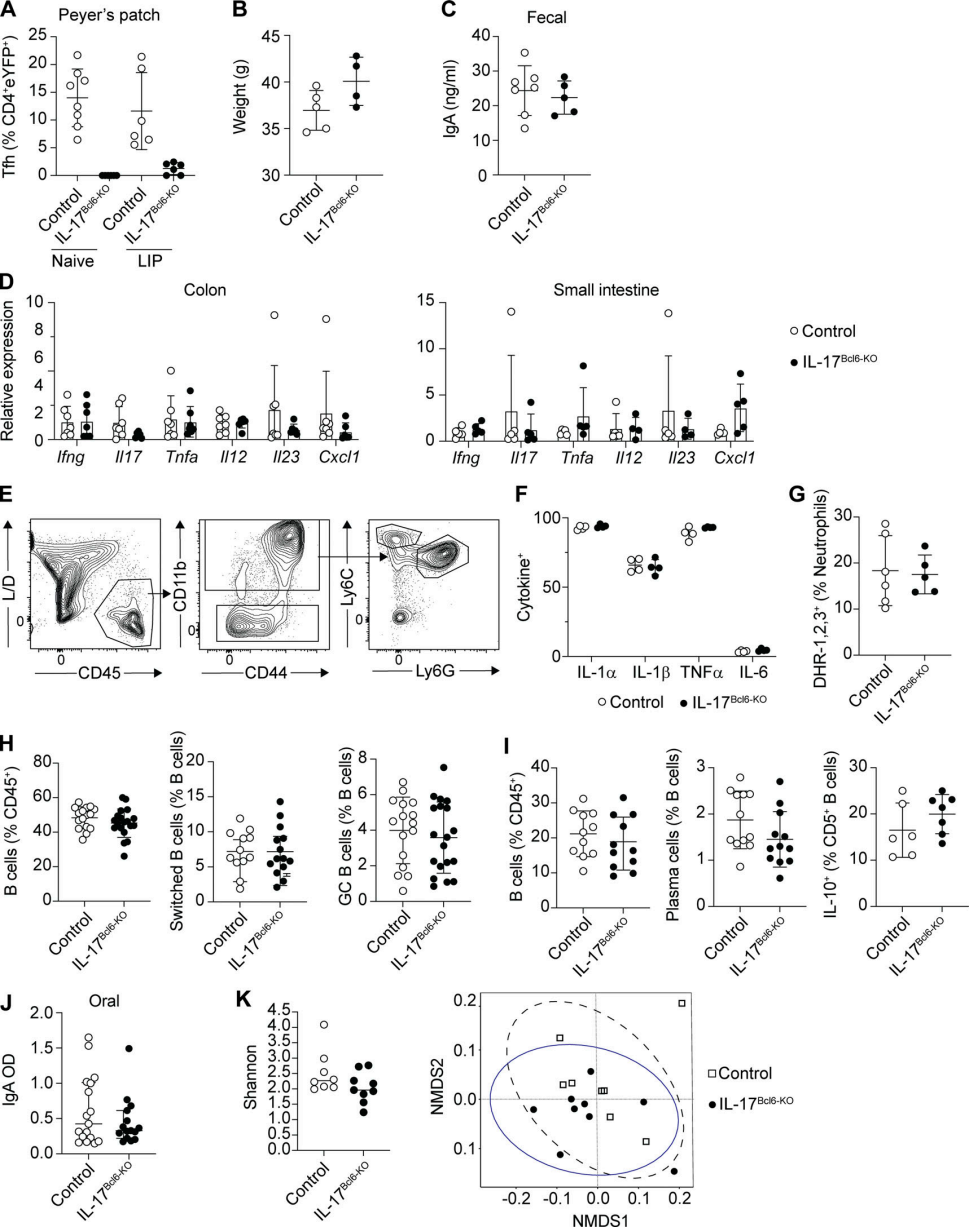
**Elevated periodontitis pathology is seen in IL-17**^**Bcl6-KO**^ **mice. (A)** LIP was induced in IL-17^Bcl6-KO^ and littermate control mice and immune parameters examined 9–10 days after ligature placement. Graph shows proportion of eYFP^+^ CD4^+^ T cells which exhibit a Tfh (CXCR5^+^PD-1^+^) phenotype in the Peyer’s patches of IL-17^Bcl6-KO^ (black symbols) and control (open symbols) mice. **(B and C)** Graphs show mouse weight and fecal IgA concentrations in adult naive IL-17^Bcl6-KO^ and control mice. Data are pooled from two to three experiments with two to four mice per group. **(D)** Relative expression of indicated genes in the colon and small intestine of IL-17^Bcl6-KO^ (black symbols) and control (open symbols) mice. Expression in IL-17^Bcl6-KO^ mice is presented relative to that in controls; data are pooled from four to six mice per group. **(E)** Representative FACS plots showing gating strategy and exemplar staining for gingival neutrophils (L/D; live/dead stain). **(F and G)** Graphs show proportions of gingival neutrophils staining positive for cytokine (F) or ROS (G) from IL-17^Bcl6-KO^ and control mice with LIP. Data are pooled from two experiments with one to three mice per group. **(H)** Graphs show proportions of total B cells, switched B cells, and GC B cells in the dLN of IL-17^Bcl6-KO^ and control mice with LIP. **(I)** Graphs show proportions of total B cells, PCs, and IL-10^+^ CD5^+^ B cells in the gingiva of IL-17^Bcl6-KO^ and control mice with LIP. Data are pooled from five to six (H) or two to four (I) experiments with two to three mice per group. **(J)** LIP was induced in IL-17^Bcl6-KO^ and control mice and IgA levels in the oral rinse examined; graph shows OD values from ELISA. Data are pooled from 15 to 17 mice from four separate experiments. **(K)** Left: Bar graph shows alpha-diversity of oral microbial communities as determined by Shannon diversity index. Right: Bray-Curtis–based non-metric multidimensional scaling (NMDS) plot of samples from control and IL-17^Bcl6-KO^ LIP animals (*n* = 8 control samples [open squares] and *n* = 9 IL-17^Bcl6-KO^ samples [black circles]). Results are expressed as mean ± SD (except for J, which is mean ± interquartile range).

We have established that Th17-to-Tfh plasticity is not a generic feature of Th17 responses, yet is a substantial component of the Th17 response in the dLN during periodontitis. Next, we wanted to understand the functional significance of this LIP-induced Th17 plasticity. To do this, we bred *Il17a*^*Cre*^*R26R*^*eYFP*^ with *Bcl6*^*fl*/fl^ (Hollister et al., 2013) mice to generate animals in which cells that have expressed IL-17 would be Bcl6 deficient, thus preventing Th17 cells from acquiring Tfh features. *Bcl6* expression was reduced in IL-17–producing cells as evidenced by a significant decrease in the proportion of eYFP^+^ cells exhibiting a Tfh phenotype in *Bcl6*^*fl/fl*^x*Il17a*^*Cre*^*R26R*^*eYFP*^ compared with littermate control mice (either *Bcl6*^*fl/+*^x*Il17a*^*Cre*^*R26R*^*eYFP*^ or *Bcl6*^*+/+*^x*Il17a*^*Cre*^*R26R*^*eYFP*^) with LIP (Fig. 5, A and B). As expected, eYFP^+^Tfh^+^ cells were also absent in the Peyer’s patches of naive and LIP *Bcl6*^*fl/fl*^x*Il17a*^*Cre*^*R26R*^*eYFP*^ mice (Fig. S3 A). These data indicate that Th17-to-Tfh plasticity was substantially restricted in *Bcl6*^*fl/fl*^x*Il17a*^*Cre*^*R26R*^*eYFP*^ (hereafter referred to as IL-17^Bcl6-KO^) animals compared with controls. Despite Th17-to-Tfh plasticity being prevented in the Peyer’s patches even at steady-state, no inflammatory phenotype was seen in the gastrointestinal tract of IL-17^Bcl6-KO^ animals, and fecal IgA levels were unchanged (Fig. S3, B–D).

We next examined LIP severity in IL-17^Bcl6-KO^ compared with co-housed, littermate controls and found that when Th17-to-Tfh plasticity was prevented, ligature-induced bone loss was elevated (Fig. 5 C). In line with this, cellularity of the dLN was higher in IL-17^Bcl6-KO^ mice compared with controls (Fig. 5 D), further indicating an augmented LIP-driven immune response in IL-17^Bcl6-KO^ mice. These data, indicate that eYFP^+^Tfh^+^ cells are not pathogenic cells but instead mediate protection and can limit development of LIP pathology.

Loss of eYFP^+^Tfh^+^ cells could result in enhanced periodontal pathology due to a concomitant increase in the proportion of conventional Th17 cells and IL-17, which we (Fig. 1) and others have shown is a driver of LIP pathology. However, examination of IL-17^+^CD4^+^ T cells demonstrated no difference in the proportion of IL-17A^+^ cells nor in the staining intensity of IL-17A per cell between IL-17^Bcl6-KO^ and control animals (Fig. 5 E), indicating that the elevated pathology seen in IL-17^Bcl6-KO^ mice was not due to enhanced proportions of conventional Th17 cells.

To begin to explore how loss of Th17-to-Tfh plasticity resulted in elevated LIP pathology, we examined the gingiva of these animals during LIP. In line with the elevated pathology, there were increased proportions of neutrophils in the gingiva of IL-17^Bcl6-KO^ compared with controls (Fig. 5 F and Fig. S3 E for exemplar staining). However, despite increased proportions, gingival neutrophils expressed similar levels of proinflammatory factors in IL-17^Bcl6-KO^ and control mice, ex vivo staining positive for cytokines and ROS (Fig. S3, F and G).

To better understand the driver of the increased inflammation seen in the IL-17^Bcl6-KO^ mice compared with controls, we first broadly profiled the B cells within the dLN and inflamed gingiva during LIP, as loss of Th17-to-Tfh plasticity could impact a number of downstream B cell activities, including but not limited to antibody production. However, following induction of LIP proportions of B lineage populations were similar in IL-17^Bcl6-KO^ mice compared with controls (Fig. S3, H and I). We next examined oral antibody levels as these could be altered in the absence of eYFP^+^Tfh^+^ cells. Surprisingly, we saw decreases in only certain antibodies in the oral rinses of IL-17^Bcl6-KO^ mice; specifically, of the antibodies that increase during LIP (Fig. 4 H), we saw a decrease in only IgG1 and IgG2c in IL-17^Bcl6-KO^ mice, with IgG2b levels remaining unchanged (Fig. 5 G). Given the previous role identified for eYFP^+^Tfh^+^ cells in IgA production in the gut (Hirota et al., 2013), we also examined the levels of this antibody in oral rinses from LIP mice, but this was unchanged in IL-17^Bcl6-KO^ mice compared with controls (Fig. S3 J). Combined, these data indicate a key role for Th17-to-Tfh plasticity in supporting production of both IgG1 and IgG2c during oral inflammation. During LIP in IL-17^Bcl6-KO^ mice, both non-Th17 Tfh and conventional Th17 remain unaffected, likely accounting for the unaffected production of IgG2b and reductions, as opposed to complete loss, in IgG1 and IgG2c. Indeed, given that eYFP^+^Tfh^+^ cells constitute only a small proportion of the total Tfh population, that the loss of these cells in IL-17^Bcl6-KO^ mice results in any alteration in oral antibody highlights the key role these cells play in ensuring effective antibody production at this site.

As the composition of antibody in the oral cavity during LIP was altered in IL-17^Bcl6-KO^ mice compared with controls, we next queried whether the exacerbated periodontitis pathology seen in IL-17^Bcl6-KO^ mice arose due to a failure to control oral microbes, vital for periodontitis pathology (Dutzan et al., 2018; Hajishengallis et al., 2011). There were no dramatic changes in the global composition of the oral bacterial community at the phylum level when comparing IL-17^Bcl6-KO^ and littermate controls with LIP (Fig. 5 H). However, minor differences were evident at the genus level in bacteria present at low abundance (Fig. 5 I), with only *Acinetobacter* elevated in IL-17^Bcl6-KO^ mice compared to controls. However, broader comparative analysis of the microbial community composition indicated no significant differences in alpha- or beta-diversity indexes in control versus IL-17^Bcl6-KO^ mice with LIP (Fig. S3 K). Despite the oral commensal community composition being largely unaltered in IL-17^Bcl6-KO^ mice with LIP relative to controls, oral bacterial loads were significantly elevated in these animals as determined by both 16S quantitative PCR (qPCR) (Fig. 5 J) and CFU counts (Fig. 5 K). Combined these data indicate a key role for eYFP^+^Tfh^+^ cells and subsequent production of IgG1 and IgG2c in restricting the biomass of the oral commensal community.

Previous work has highlighted vital pathogenic roles for Th17 cells in periodontitis. In contrast, here, we outline an important pathway by which a subset of Th17 cells acquire a Tfh cell phenotype and demonstrate that this Th17-to-Tfh diversification is important in gingival barrier defense. This is distinct from the role of Bcl6-expressing Th17 cells in EAE, where Bcl-6^+^Th17 cells are thought to promote development of pathogenic tertiary lymphoid structures, B cell class switching, and tissue inflammation (Hartlehnert et al., 2021; Peters et al., 2011). During periodontitis, we show that eYFP^+^Tfh^+^ cells mediate non-inflammatory roles as in their absence elevated pathology is not simply a result of increased proportions of conventional Th17 cells. Instead, we see reduction in antibody levels within the oral cavity. We saw no change in oral IgA levels, although the majority of this isotype is generated by PC in the salivary glands (Brandtzaeg et al., 1991; Zubeidat et al., 2023). In contrast, we do see changes in specific IgG isotypes in the oral cavity of IL-17^Bcl6-KO^ mice.

Oral IgG is largely thought of as contributing to pathology in periodontitis as depositions of IgG are seen in the gingiva during disease (Nikolopoulou-Papaconstantinou et al., 1987; Toto et al., 1978), as are increased frequencies of IgG^+^ PC (Caetano et al., 2021; Williams et al., 2021). Importantly, our data also suggest barrier defensive, as opposed to tissue destructive, roles for some oral IgG during periodontitis. In fact, IgG responses to oral bacteria are seen in healthy patients; they are just elevated during periodontitis (Ebersole et al., 2021; Sweier et al., 2009). The specificity of tissue-protective IgG remains to be defined and will be important to determine, but given the similarity of the oral commensal community in IL-17^Bcl6-KO^ and control mice with LIP, it appears a key role for oral IgG could be to more broadly limit the oral bacterial biomass.

Our study provides a greater granularity to previous work examining the role of B cells in periodontitis, where B cells have been shown to promote periodontal pathology. In murine studies, periodontitis severity is reduced in the absence of B cells (Abe et al., 2015; Oliver-Bell et al., 2015). In humans, anti-CD20 treatment (which depletes B cells) leads to improvements in clinical periodontal parameters (Coat et al., 2015). Together with our data, these studies suggest that pathogenic roles for B cells can now be uncoupled from protective roles for B cells during periodontitis, with production of some IgG1 and IgG2c being important in limiting disease pathology. As such we can begin to provide a more refined understanding of the role B lineage cells mediate during oral inflammation. In this way, our work highlights that it is now important to better elucidate the pattern of antibody isotype production during periodontitis, as well as its specificity, and determine how this contributes to constraint of commensal microbes versus tissue destruction.

Combined, our data outline a differentiation pathway for Th17 cells during periodontitis, detailing a Th17-to-Tfh plasticity that is dependent upon IL-6. Our data demonstrate a key role for Th17-to-Tfh cells in limiting periodontitis pathology, establishing that a degree of Th17 cell plasticity is vital to safeguard effective gingiva defense and integrity during oral inflammation.

## Materials and methods

### Mice

All mice were bred at the University of Manchester. Mice aged between 10 and 28 wk were housed in individually ventilated cages, under specific pathogen–free conditions, with standard chow and water provided ad libitum, at a constant temperature and a 12-h light and dark cycle. C57BL/6 *Il17a*^Cre^*Rosa26*^eYFP^ mice were originally generated by Prof B. Stockinger (Hirota et al., 2011). For *Il17a-*KO experiments, C57BL/6 *Il17a*^Cre^*Rosa26*^eYFP^ heterozygous mice were bred to generate *Il17a*^Cre-Homozygous^*Rosa26*^eYFP^ mice and non cre-homozygous controls. *Bcl6*^fl/fl^ mice were originally generated by Prof A. Dent (Hollister et al., 2013) and *Tgfbr2*^fl/fl^ by Prof S. Karlsson (Levéen et al., 2002); these were bred with *Il17a*^Cre^*Rosa26*^eYFP^ mice. Mice were age- and sex-matched within experiments, but both males and females were used. All experiments were approved by the University of Manchester Animal Welfare Ethical Review Body and the Home Office, UK, and performed following local rules.

### LIP

Bone loss was induced through the use of a 5-0 silk ligature tied around the maxillary second molars, with the ligature placed in the gingival sulcus as previously described (Krishnan et al., 2018); this treatment induces alveolar bone loss, which was measured 9–10 days after ligature placement. In some experiments blocking antibodies, anti-IL6 (clone MP5-20F3; BioXCell), anti-IFNAR (MAR1-5A3; BioXCell), or isotype control (IgG1, clone HPRN; BioXCell) were administered. Periodontal bone heights were assessed after defleshing and staining with methylene blue. The distance between the CEJ and ABC (CEJ-ABC distance) was measured at six predetermined sites.

### *C. rodentium* infection

*C. rodentium* (nalidixic acid-resistant strain) was originally a gift from Prof. G. Frankel (Imperial College London, London, UK). A single colony of *C. rodentium* was transferred to Luria-Bertani (LB) broth containing nalidixic acid (50 μg/ml) and grown for 14–16 h at 37°C in a shaking incubator. *Il17a*^Cre^*Rosa26*^eYFP^ mice were gavaged with 200 μl of PBS containing ∼2 × 10^9^ CFU. Serial CFU dilutions of *C. rodentium* were used to determine bacterial load. Animals were euthanized 14 days after infection and their mesenteric lymph nodes harvested.

### AIA

AIA, driven by methylated bovine serum albumin (mBSA) antigen, was induced as previously described (Jones et al., 2018). Briefly, 8–14-wk-old *Il17a*^Cre^*Rosa26*^eYFP^ mice were immunized on day −21 and −14 with 2 mg/ml mBSA (Sigma-Aldrich) in 4 mg/ml complete Freund’s adjuvant (MD Bioscience) by subcutaneous injection to the right-hand then left-hand flank. At day −21 mice also received an i.p. injection of 1.6 μg/ml Pertussis toxin (Sigma-Aldrich). On day 0 an intra-articular injection of 10 μl of 10 mg/ml mBSA was administered to the left knee. Mice were euthanized at peak of inflammation at day 3 and popliteal lymph nodes harvested.

### IMQ-induced skin inflammation

*Il17a*^Cre^*Rosa26*^eYFP^ were treated with 10 mg of Aldara Cream on each ear pinnae and back skin for 6 consecutive days under anesthesia before ear-draining lymph nodes were harvested.

### Preparation of single-cell suspensions

#### Gingiva

Mouse gingiva were dissected as previously described (Dutzan et al., 2016). Tissue blocks were dissected and digested for 30 min at 37°C in RPMI 1640 (Sigma-Aldrich) supplemented with 2 mM *L*-glutamine (Sigma-Aldrich), 10 mM Hepes (Sigma-Aldrich), 3.2 mg/ml collagenase IV (Gibco), and 7.5 µg/ml DNase (Sigma-Aldrich). Following removal of gingiva tissue from maxilla and mandible, single-cell suspensions were obtained by mashing tissues through a 70-µm cell strainer.

#### Spleen and lymph nodes

Spleens and lymph nodes were mashed through a 70-µm strainer. For spleens, red blood cells were lysed by incubation with ammonium-chloride-potassium lysing buffer (Lonza) for 3 min on ice.

### Ex vivo cell stimulation

For assessment of T cell cytokine production, cells were stimulated with eBioscience Cell Stimulation Cocktail in the presence of GolgiPlug (Brefeldin A; BD Biosciences). After 3–3.5 h, cells were stained for flow cytometric analysis. For assessment of B cell production of IL-10, cells were stimulated with eBioscience Cell Stimulation Cocktail containing protein transport inhibitors plus 10 µg/ml LPS for 5 h, after which they were stained for flow cytometric analysis. For assessment of neutrophil cytokine production, cells were cultured in media alone with GoligiPlug for 2.5 h and then stained for flow cytometric analysis. For assessment of ROS production, cell preparations were stained for 15 min at 37°C with 75 ng/ml DHR-123 (Sigma-Aldrich) prior to staining for flow cytometric analysis.

### Flow cytometry

Single-cell suspensions from tissues were washed in PBS and incubated with antibody cocktails in FACS buffer (PBS with 2% FCS and 2 mM EDTA) plus anti-CD16/32 (2.4G2; BioXcell) for 15 min at 4°C. Cell were stained with combinations of the following antibodies, which were obtained from eBioscience and BioLegend: B220 (RA3-6B2), Ly6G (1A8), Ly6C (HK1.4), CD11b (M1/70), CD11c (N418), MHCII (M5/114.15.2), CD138 (281-2), CD185 (L138D7), CD19 (ID3), CCR6 (29-2L170), CD279 (29F.1A12), CD38 (90), TCRβ (H57-597), CD4 (RM4-5), CD5 (53-7.3), CD44 (1M7), CD43 (S7), CD45 (30-F11), CD8 (53-6.7), CD95 (SA367H8), Foxp3 (FJK-16s), GL-7 (GL7), IgA (RMA-1), IgD (11-26c2a), IgG (Poly4053), IgG1 (RMG1-1), IgM (RMM-1), Foxp3 (FJK-16s), RORγt (B2D), Bcl6 (K112-91), GMCSF (MP1-22E9), IFNγ (XMG1.2), IL-10 (JES5-16E3), IL-17A (TC11-18H10.1), IL-22 (Poly5164), TNFα (MP6-XT22), pro-IL-1β (NJTEN3), IL-1α (ALF-161), and IL-6 (MP5-20F3). Dead cells were excluded by use of a Live/Dead Fixable blue dead cell stain kit (Molecular Probes), Zombie UV, or Aqua Fixable Viability Kit (BioLegend). When biotin-conjugated antibodies were used, cells were washed and stained with fluorochrome-conjugated streptavidin (BioLegend) for 15 min at 4°C. Following surface staining, cells were fixed in Fixation and Permeabilization Solution (BD Cytofix/Cytoperm) for 1 h at 4°C in the dark prior to intracellular staining to maintain eYFP expression. For intracellular staining, samples were stained overnight at 4°C in the dark with fluorochrome-conjugated antibodies in eBioscience Foxp3 Permeabilization Buffer. Samples were acquired using a BD LSRFortessa or FACSymphony (BD Biosciences) flow cytometer and analyzed with FlowJo (Treestar).

### TCR sequencing

Amplification of mouse TCR α chains from ∼40 sorted cells per well was performed by iRepertoire Inc. using a modification of iRepertoire’s single-cell amplification technology, referred to as iPair mini-bulk. Briefly, reverse transcription PCR round 1 (RT-PCR1) was performed with nested, multiplex primers and included partial Illumina adaptors. Included on the reverse primer is an in-line six-nucleotide barcode, which serves as a plate identifier so that multiple 96-well plates can be multiplexed in the same sequencing flow cell. After RT-PCR1, the first round of PCR1 products were rescued using SPRISelect Beads (Beckman Coulter). A second PCR was performed with dual-indexed primers that complete the sequencing adaptors introduced during PCR1 and provide plate positional information for the sequenced products. Sequencing was performed using the Illumina MiSeq 500-cycle Nano kit with 281 × 221 cycles paired-end read. Raw data were demultiplexed by Illumina dual indices and the six-nt internal plate barcode information for each well of the 96-well PCR plates. Due to the limited number of cells, the iRepertoire single-cell pipeline “atlas” was used. First, low-quality sequences are filtered with “fastp.” For each sequence, the c-gene is called using a Smith-Waterman algorithm and V and C primers are trimmed from the ends of the read. A PCR-error filter is then applied to cluster sequences into “clones” consisting of a dominant sequence and its PCR artifacts using a clustering threshold, and child clones are removed if below a parent/child ratio, with their counts consolidated into their parent clone’s count. For the few remaining sequences, a custom program is used to infer the missing V germline sequence and translate it with the appropriate reading frame. Sequences are then mapped and annotated using “ANARCI.” Finally, to account for “dual index hopping” in Illumina sequencers, a filter is applied whereby sequences are removed if they share a percent similarity and have <2% of the reads of another well of the same row or column in the run.

### ELISA of oral rinses

Oral rinses were obtained by washing the oral cavity with 50 μl of PBS which was then stored for downstream applications. ELISA kits were purchased from Invitrogen and utilized as per the manufacturer’s instruction to quantify levels of IgA, IgG, IgG1, IgG2b, IgG2c, and IgG3. Plates were read on a Tecan Infinite M200 PRO microplate reader running I-control 1.9 software.

### qPCR

Total RNA was obtained from tissues using Trizol and cDNA was synthesized using Superscript reverse transcription kit (Invitrogen/Life Technologies). Quantitative real-time PCR was done with SYBR green qPCR super mix (Invitrogen/Life Technologies) and normalized to *hprt* expression. The following primers were used: *ifng* (forward: 5′-ATG​AAC​GCT​ACA​CAC​TGC​ATC-3′, reverse: 5′-CCA​TCC​TTT​TGC​CAG​TTC​CTC-3′), *il17a* (forward: 5′-TTT​AAC​TCC​CTT​GGC​GCA​AAA-3′, reverse: 5′-CTT​TCC​CTC​CGC​ATT​GAC​AC-3′), *il23a* (forward: 5′-ATG​CTG​GAT​TGC​AGA​GCA​GTA-3′, reverse: 5′-ACG​GGG​CAC​ATT​ATT​TTT​AGT​CT-3′), *cxcl1* (forward: 5′-CTG​GGA​TTC​ACC​TCA​AGA​ACA​TC-3′, reverse: 5′-CAG​GGT​CAA​GGC​AAG​CCT​C-3′), *tnfa* (forward: 5′-CCC​TCA​CAT​CAG​ATC​ATC​TTC​T-3′, reverse: 5′-GCT​ACG​ACG​TGG​GCT​ACA​G-3′), and *il12* (forward: 5′-CAT​CGA​TGA​GCT​GAT​GCA​GT-3′, reverse: 5′-CAG​ATA​GCC​CAT​CAC​CCT​GT-3′).

### Bacterial 16S qPCR

The murine oral cavity was sampled for 30 s using sterile ultrafine swabs as previously described (Abusleme et al., 2017). Bacterial DNA was then isolated from these swabs using the DNeasy Powersoil Kit (Qiagen). Bacterial DNA samples were amplified using the following primers for total 16S forward; 5′-ACT​CCT​ACG​GGA​GGC​AGC​AGT-3′ and reverse; 5′-ATT​ACC​GCG​GCT​GCT​GGC-3′. 16S copy number was determined by running a standard curve with a known 16S copy number.

### 16S sequencing and analysis of the oral microbiome

DNA was extracted from oral swabs as for 16S qPCR. Amplicon generation, sequencing, and bioinformatics analysis were undertaken by Novogene (China). Briefly, the V4 16S ribosomal RNA (rRNA) region was amplified and amplicons sequenced on an Illumina platform and 250-base-pair paired-end reads were generated. Data filtration and chimera removal were done using Fastq and Vsearch, and then amplicon sequence variants (ASVs) were generated from the raw data using the dada2 pipeline (Callahan et al., 2016). Subsequently, by applying QIIME2’s classify-sklearn algorithm (Bokulich et al., 2018; Bolyen et al., 2019), a pretrained Naive Bayes classifier was used for species annotation of each ASV, utilizing the Silva 138.1 database. This allowed abundance tables at the level of kingdom, phyla, class, order, family, genus, and species to be obtained. Differences in community diversity and structure were determined by calculating alpha- and beta-diversity indices with QIIME2 and R.

### Bacterial CFU assessment

The murine oral cavity was sampled as above with sterile ultrafine swabs that were placed in Eppendorf tubes with 200 µl of sterile PBS. Swabs were vortexed prior to bacterial isolation and serial dilutions of resultant bacterial suspensions plated onto LB (Sigma-Aldrich) or Wilkins-Chalgren (Sigma-Aldrich) agar plates. CFU was enumerated following anaerobic (Wilkins-Chalgren) or aerobic (LB) growth at 37°C.

### Statistics

Statistical analyses were performed using GraphPad Prism. The use of parametric or non-parametric tests was decided based on normal distribution of the data points, determined using Shapiro-Wilk test. When two groups were compared, a Student’s *t* test for normal-distributed data, or a Mann–Whitney test for non-parametric analysis were employed. When comparing more than two groups, a one-way ANOVA with Dunnet’s multiple comparison analysis for normal-distributed data or a Kruskal–Wallis test with Dunn’s multiple comparison test for non-parametric analysis was employed. For analysis of TCR repertoire, all cells related to individual samples were pooled into single clonesets. The clonal sizes were established by grouping the clones using peptide-unique CDR3 sequences. Then, the richness, diversity, and similarity of the resulting sequences were estimated using common repertoire statistics (Chiffelle et al., 2020). The repertoire richness was defined as a number of unique CDR3 sequences adjusted for the cell input. For similarity analysis, the exact number of shared sequences, the fraction of shared sequences, and the fraction of shared reads (Horn’s index) between all samples were calculated. Then, the relative abundance of shared sequences between two cell populations of each mouse was generated using similarity results.

### Online supplemental material

Fig. S1 shows the predominant production of IL-17A by gingival Th17 cells during periodontitis. This figure also shows proportions of eYFP^+^Tfh^+^ cells in the Payer’s patches and non-draining lymph nodes as well as their phenotype in the gingiva dLN. Fig. S2 shows the proportions of eYFP^+^ and total Tfh in the lymph nodes draining sites of inflammation following *C. rodentium* infection, IMQ-induced psoriasis, and antigen-induced arthritis. This figure also shows example FACS plots gating different B lineage populations. Fig. S3 outlines the phenotypic details of the IL-17^Bcl6-KO^ mice, showing no inflammation in the gastrointestinal tract of naive mice and gingival neutrophil phenotypes, B cell phenotypes, and oral IgA levels. Finally, this figure outlines the broad similarity of the oral commensal community in IL-17^Bcl6-KO^ mice compared with controls.

## Data Availability

Data are available in the article itself and its supplementary materials and are also available upon request from the corresponding author.
